# HPV16 E6E7 up-regulates KIF2A expression by activating JNK/c-Jun signal, is beneficial to migration and invasion of cervical cancer cells

**DOI:** 10.1515/med-2022-0578

**Published:** 2022-11-14

**Authors:** Yuyan Wang, Jinfeng Wang, Anqi Zhao, Xin Huang, Xin Zhang

**Affiliations:** Department of Obstetrics and Gynecology, The First Affiliated Hospital of Jinzhou Medical University, Jinzhou, Liaoning 121000, China; Department of Pediatrics, The First Affiliated Hospital of Jinzhou Medical University, Jinzhou, Liaoning 121000, China; Department of Obstetrics and Gynecology, Xuanwu Hospital Capital Medical University, Beijing 100053, China; Department of Oncology, The First Affiliated Hospital of Jinzhou Medical University, Jinzhou, Liaoning 121000, China

**Keywords:** cervical cancer, HPV, E6/E7, KIF2A, JUN

## Abstract

Cervical cancer is the fourth most common cancer and the fourth leading cause of cancer death in women. Human papillomavirus (HPV16) E6/E7 heterogenous expression in C33A cells increased the mRNA and protein levels of KIF2A, while siRNA deletion of endogenous E6/E7 reduced the mRNA and protein levels of KIF2A in SiHa cells. KIF2A promoted cell migration and invasion, and regulated the expression of epithelial–mesenchymal transition-related proteins in C33A and SiHa cells. The exogenous expression of E6/E7 in C33A cells increased the phosphorylation of Akt, ERK, and JNK. However, Akt (API-2) and ERK (PD98059) inhibitors had no effect on the increase in KIF2A expression induced by E6/E7, while JNK inhibitors (JNK-IN-8 and SP600125) blocked the increase in KIF2A expression induced by E6/E7. The exogenous expression of E6/E7 increased the levels of transcription factor c-Jun, which is the classic substrate of JNK. Knockdown of c-Jun reduced the increase in KIF2A expression induced by E6/E7. In summary, KIF2A plays a key role in the motility and metastasis of cervical cancer. HPV16 E6/E7 can increase the levels of transcription factor c-Jun by activating the JNK signal, thereby up-regulating the transcriptional expression of KIF2A.

## 1 Introduction

Cervical cancer is the fourth most common cancer and the fourth leading cause of cancer death in women, according to the 2018 Global Cancer Estimates [1]. There were an estimated 570,000 cases and more than 310,000 deaths worldwide in 2018 [1]. In addition, cervical cancer survival has not improved significantly since 1975 [2]. High-risk human papillomavirus (HPV) infection is actually a necessary cause of cervical cancer, especially HPV-16 [3]. In fact, 79–100% of invasive cervical cancer cases worldwide are associated with DNA from high-risk HPV types, approximately 70% of which are associated with HPV-16 and HPV-18 [4]. The development of cervical cancer depends on the continued expression of viral oncogenes E6 and E7, which together act to transform host cells. The most well-known targets of E6 and E7 are p53 and pRb, respectively, to disrupt DNA repair and cell cycle regulation [5,6]. However, they are not the only target.

The human kinesin-13 family consists of KIF2A, KIF2B, KIF2C/MCAK, and KIF24 [7]. Different from the traditional kinesin involved in the transport of intracellular substances, the kinesin-13 protein does not “walk” along the microtubules (MTs), but triggers the depolymerization of MT [7,8] which plays a role in a series of physiological environments, such as spindle assembly, chromosome separation, axon growth, and cilia disintegration [9]. Therefore, KIF2A is also considered to be a MT depolymerase [10]. The latest research shows that AMPPNP-bound KIF2A can form a stable complex with tubulin and trigger MT depolymerization [8]. In 2020, Hu et al. found that the mRNA and protein expression of KIF2A in cervical cancer tissues was higher than that in the adjacent tissues [11]. The expression level of KIF2A in tumor tissues is positively correlated with lymph node metastasis and FIGO (Federation International of Gynecology and Obstetrics) staging, and is closely related to disease-free survival and overall survival of the patients [11]. In addition, the expression of KIF2A in lung cancer cells also increased significantly [12]. Some studies believe that the expression of KIF2A increases the dynamic instability of MTs, thereby supporting tumor cell migration and invasion [12]. Loss of KIF2A can impair the ability of mutant K-Ras transformed cells to migrate and invade the matrix gel [12].

During cervical cancer progression, the c-Jun N-terminal kinases (JNK) signaling pathway is activated [13]. c-Jun is an important downstream substrate of JNK, and is often associated with tumorigenesis [14]. Blocking JNK signaling with small-molecule inhibitors or knocking down the JNK substrate c-Jun inhibits cervical cancer cell proliferation and induced apoptosis [13]. JNK/c-Jun signaling can also promote the invasive potential of cervical cancer cells, and is required for the expression of the epithelial–mesenchymal transition (EMT)-related transcription factor Slug and the mesenchymal marker vimentin [13]. Furthermore, the HPV E6 oncogene induces JNK1/2 phosphorylation in a manner that requires the E6 PSD95/DLG1/ZO1 (PDZ)-binding motif [13].

Therefore, this study will investigate the role of KIF2A in the migration and invasion of cervical cancer cells, and the molecular mechanism of E6/E7 protein regulating the transcription and expression of KIF2A by activating the JNK signaling pathway.

## 2 Methods

### 2.1 Cell culture

C33A cells are HPV16 negative, and SiHa cells contain 1–2 integrated copies of HPV16. The human cervical cancer cell lines C33A and SiHa were purchased from ATCC, cultured in DMEM with 10% (v/v) FBS at 37°C with 5% CO_2_.

### 2.2 Cell transfection and processing

GFP-E2, GFP-E6/E7, pcDNA3.1-KIF2A, and pcDNA3.1-c-Jun overexpression plasmids, as well as siRNAs specifically targeting E2, E6/E7, KIF2A, and c-Jun, were purchased from Jikai Gene (Shanghai, China), and transfected into cells by using lipofectamine 2000 (Invitrogen). Cells transfected with empty vector served as negative controls for each experiment. 2 μM API-2 (triciribine), 10 µM PD98059, 10 µM JNK-IN-8 (covalent), and 10 µM SP600125 were purchased from Calbiochem (San Diego, CA, USA), and treated cells for 24 h.

### 2.3 RT-qPCR

Total RNA was prepared by using TRIzol reagent (Sigma), and cDNA was synthesized using the PrimeScript™ cDNA synthesis kit (Takara Bio). The cDNA samples were analyzed using the SYBR^®^ Premix Ex Taq™ (Takara Bio) on Bio-Rad cyclers (Bio-Rad, Hercules, CA, USA). Relative gene expression was normalized to the GAPDH, and calculated using the 2^−ΔΔCT^ method. The KIF2A primer sequences were forward, 5′-GCCGAATACATCAAGCAAT-3′ and reverse, 5′-CTCTCCAGGTCAATCTCTT-3′. GAPDH primer sequences were 5′-AATCCCATCACCATCTTCCAG-3′ and 5′-TCATGAGTCCTTCCACGATACC-3′.

### 2.4 Western blot

Cells were lysed with RIPA lysis buffer containing protease inhibitor cocktail (GenDEPOT, Harris County, TX, USA) for 20 min on ice. The lysate was centrifuged at 12,000 rpm for 30 min at 4°C. The protein concentrations were measured using a BCA assay kit (Thermo Fisher Scientific, Waltham, MA, USA). The lysates were separated by SDS-PAGE and transferred to a PVDF membrane (Millipore, Billerica, MA, USA). The membrane was incubated with 5% skim milk at room temperature for 1 h, then incubated with primary antibodies overnight at 4°C. After washing extensively, the membrane was incubated with HRP-conjugated secondary antibody for 2 h. The signal was detected using enhanced chemiluminescence system.

The primary antibody information used in this study is as follows: anti-KIF2A (ab197988), anti-GAPDH (ab8245), anti-N-cadherin (ab18203), anti-p-Akt (ab38449), anti-Akt (ab8805), anti-p-ERK (ab79483), anti-ERK (ab184699), anti-p-JNK (ab4821), anti-JNK (ab112501), anti-p-c-Jun (ab32385), and anti-c-Jun (ab40766) were purchased from Abcam; anti-E-cadherin (60335-1-Ig) and anti-Vimentin (10366-1-AP) were purchased from Proteintech.

### 2.5 Transwell

For migration assay, 5 × 10^3^ cells were seeded in 100 µL serum-free medium in a Transwell chamber (24-well insert; pore size, 8 µm; Corning Incorporated). About 600 µL medium containing 2% FBS was added to the lower chamber. After incubating for 24 h, the cells in the upper chamber were removed with PBS and cotton swabs. The cells in the lower chamber were fixed in 2.5% glutaraldehyde for 20 min and stained with crystal violet for 15 min at room temperature. The cells were observed under an inverted light microscope (Leica MicroSystems GmbH), and the number of cells were counted in three randomly selected fields of view at 100× magnification (Olympus Corporation). For the invasion assay, the upper surface of the chamber was coated with Matrigel.

### 2.6 Dual luciferase

The reporter plasmid expressing firefly luciferase (pGL3 Basic, Promega) under the control of c-Jun response element and c-Jun overexpression plasmid were co-transfected into cells. After 24 h of incubation, the samples were lysed in passive lysis buffer (Promega, USA), and the activity was measured using the dual luciferase reporter gene detection system (Promega).

### 2.7 Statistical analysis

All data were displayed as mean ± standard deviation of three replicates. The difference was evaluated by Student’s *t*-test using GraphPad Prism 8. It was significant at *P* < 0.05.

## 3 Results

### 3.1 KIF2A facilitates migration and invasion of C33A and SiHa cells

**Figure 1:**
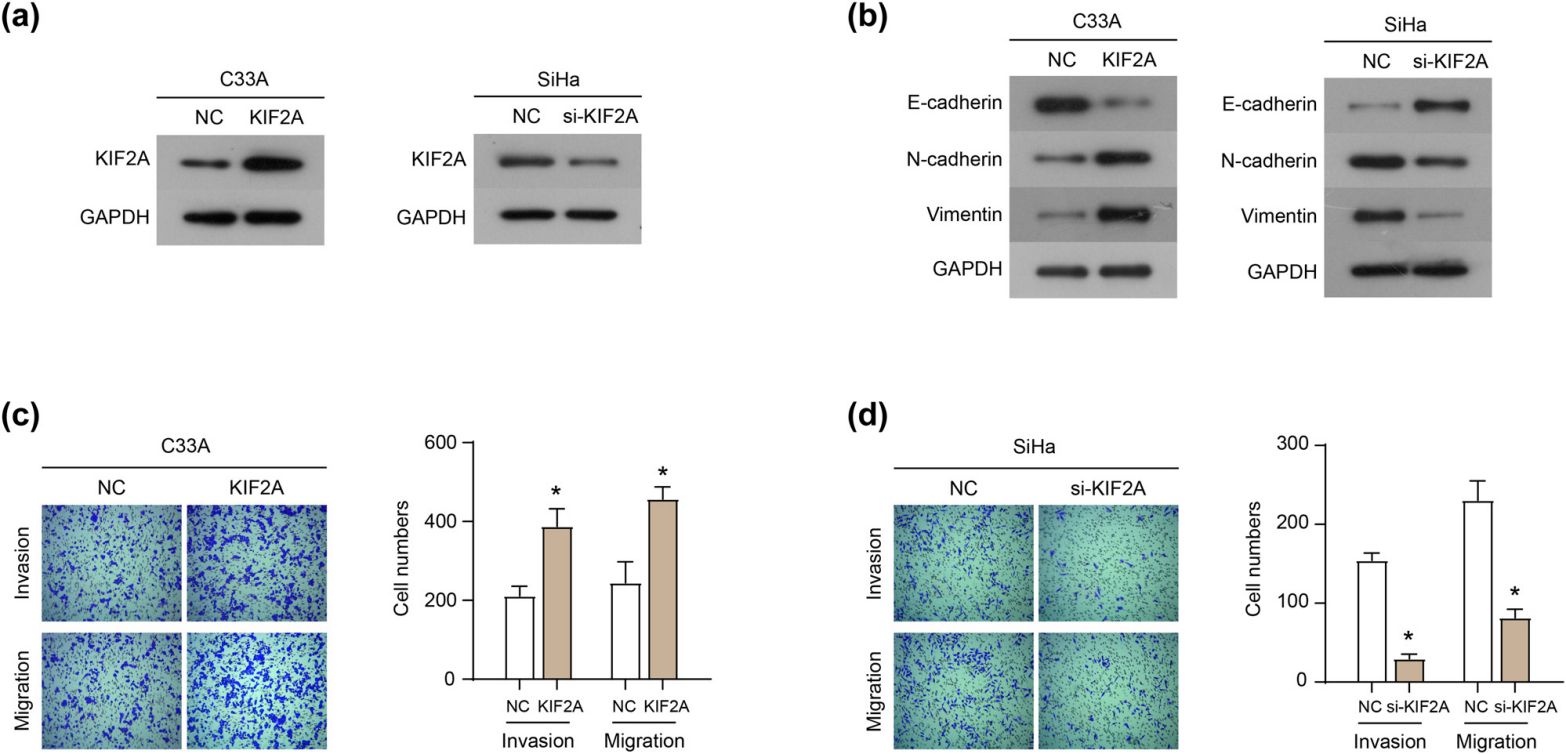
KIF2A facilitates migration and invasion of C33A and SiHa cells. (a) Expression of KIF2A in C33A and SiHa cells were overexpressed and knocked down, respectively. (b) Expression of EMT-related proteins in C33A and SiHa cells. The invasion and migration of C33A (c) and SiHa (d) cells. *P < 0.05.

The expression of KIF2A was overexpressed and knocked down in C33A and SiHa cells, respectively (Figure 1a). The exogenous expression of KIF2A in C33A cells reduced the expression of the epithelial marker E-cadherin and increased the expression of mesenchymal markers N-cadherin and Vimentin (Figure 1b). In contrast, the down-expression of KIF2A in SiHa cells increased the expression of E-cadherin and decreased the expression of N-cadherin and Vimentin (Figure 1b). This result suggests that the KIF2A expression level is associated with the EMT process of cervical cancer cells. In addition, transwell experiment showed that the exogenous expression of KIF2A in C33A cells promoted cell migration and invasion (Figure 1c), while the down-expression of KIF2A in SiHa cells inhibited cell migration and invasion (Figure 1d).

### 3.2 HPV16 E6/E7 up-regulates KIF2A expression

**Figure 2:**
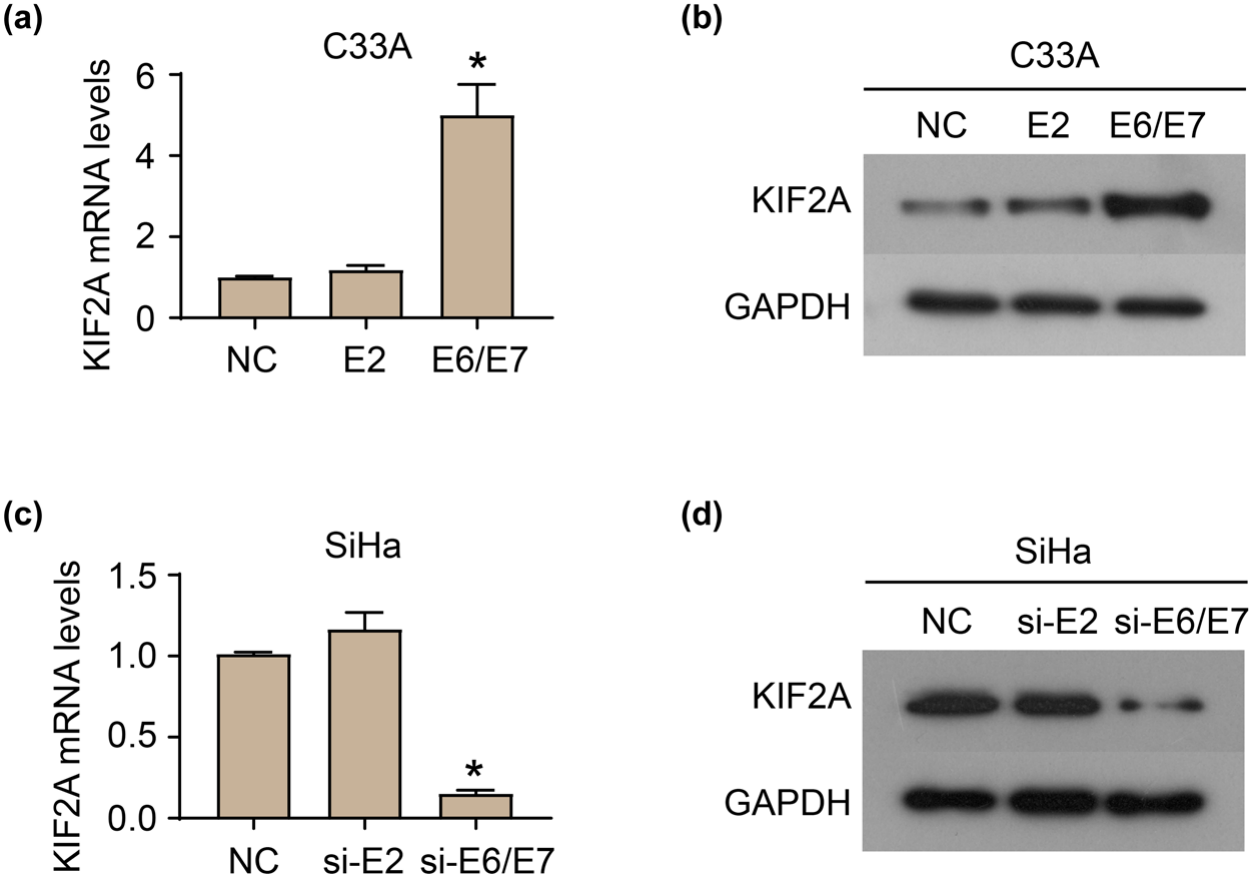
HPV16 E6E7 up-regulates KIF2A expression. mRNA (a) and protein (b) levels of KIF2A in C33A cells heterogenous expressed HPV E6E7. mRNA (c) and protein (d) levels of KIF2A in SiHa cells which endogenous E6/E7 was reduced by siRNA deletion. *P < 0.05.

The exogenous expression of HPV16 E6/E7 in C33A cells increased the mRNA and protein levels of KIF2A (Figure 2a and b). Furthermore, the siRNA deletion of endogenous E6/E7 reduced the mRNA and protein levels of KIF2A in SiHa cells (Figure 2c and d). In addition, the expression of E2 did not affect KIF2A expression in C33A and SiHa cells (Figure 2).

### 3.3 HPV16 E6/E7 up-regulates KIF2A expression by activating JNK

**Figure 3:**
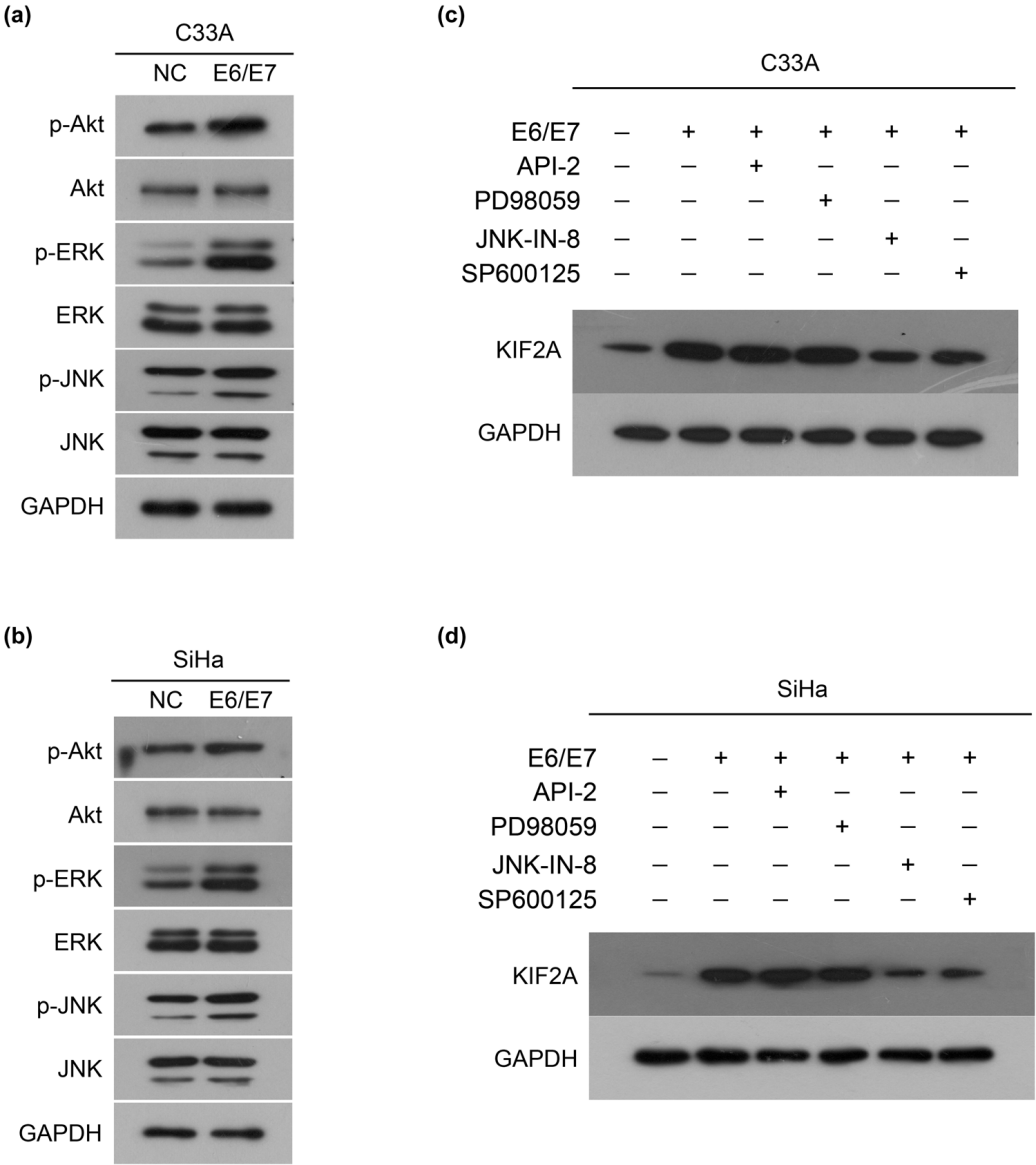
HPV16 E6E7 up-regulates KIF2A expression by activating JNK. Exogenous expression of E6/E7 in C33A (a) and SiHa (b) cells increased the phosphorylation of Akt, ERK, and JNK. Effect of Akt, ERK, and JNK inhibitors in KIF2A expression induced by E6/E7 in C33A (c) and SiHa (d) cells.

To continue to explore how E6/E7 oncogenes up-regulate KIF2A, we examined the activation of key signaling pathways, Akt, ERK, and JNK. As shown in Figure 3a and b, exogenous expression of E6/E7 in C33A and SiHa cells increased the phosphorylation of Akt, ERK, and JNK (Figure 3a). However, Akt (API-2) and ERK (PD98059) inhibitors had no effect on the increase in KIF2A expression induced by E6/E7, while JNK inhibitors (JNK-IN-8 and SP600125) blocked the increase in KIF2A expression induced by E6/E7 (Figure 3c and d). That is, activation of JNK is required for E6/E7 to up-regulate KIF2A expression.

### 3.4 JNK substrate c-Jun drive KIF2A transcription

**Figure 4:**
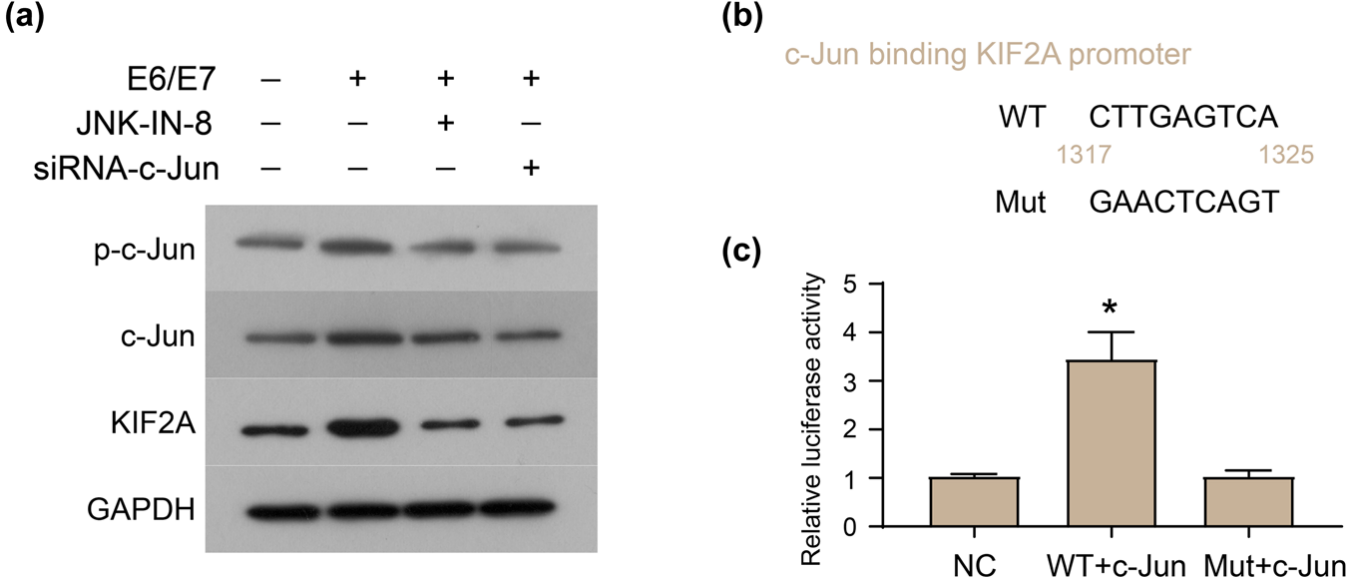
JNK substrate c-Jun drive KIF2A transcription. (a) Exogenous expression of E6/E7 in C33A cells increased the levels of c-Jun and p-c-Jun, and the JNK inhibitor (JNK-IN-8) and siRNA specifically targeting c-Jun reduced the expression levels of c-Jun, p-c-Jun, and KIF2A induced by E6/E7. (b) PROMO was used to predict the site where c-Jun binds to the KIF2A promoter sequence. (c) Dual luciferase experiment was performed. *P < 0.05.

The exogenous expression of E6/E7 in C33A cells increased the levels of c-Jun and p-c-Jun, which is classic substrate of JNK (Figure 4a). Both inhibition of JNK signaling and knockdown of c-Jun decreased the levels of c-Jun and p-c-Jun, and neutralized the expression of KIF2A up-regulated by E6/E7 (Figure 4a). That is, activation of c-Jun is required for E6/E7-induced KIF2A expression. In addition, PROMO [15] was used to predict the site where c-Jun binds to the KIF2A promoter sequence (Figure 4b), and a dual luciferase experiment was performed (Figure 4c). The results showed that c-Jun initiates the transcription of KIF2A by binding to the putative site (Figure 4c).

## 4 Discussion

KIF2A, an MT depolymerase, is found to be highly expressed in cervical cancer, and closely related to the prognosis of cervical cancer [11]. A team believes that high expression of KIF2A increases the dynamic instability of MT, thereby supporting cell migration and invasion [12]. However, there is no exact experiment to verify the role of KIF2A expression in cervical cancer cell motility. The results of transwell experiments in this study showed that high expression of KIF2A promoted the migration and invasion of cervical cancer, and low expression of KIF2A hindered the migration and invasion of cervical cancer. The results of western blot also indicated that the expression of KIF2A was closely related to the EMT process. These results suggest an important role of KIF2A expression in cervical cancer motility, although *in vivo* experiments are still lacking. The regulation of MT dynamics by KIF2A can affect the activation of signaling pathways that affect the cell behavior more broadly. For example, Elma et al. found that KIF2A can regulate the localization of lysosomes in the cytoplasm, thereby activating the mTORC1 signaling on the lysosomal membrane [16]. Furthermore, KIF2A appears to activate the PI3K/Akt signaling pathway in lung cancer and gastric cancer [17–20].

High-risk HPV infection is associated with the development of cervical cancer. The continuous expression and regulation of viral E6/E7 oncoprotein integrated into the host cell is necessary for initiation and maintenance of the transformed phenotype. The malignant progression of HPV-related precancerous lesions into malignant tumors is a rather slow and rare event. In addition to interfering with key regulatory pathways, namely p53 and pRb tumor suppressor pathways, high-risk HPV E6/E7 proteins must also regulate many key molecules. For example, study has found that HPV16 E7 can be associated with β-tubulin in primary human cells to induce abnormal centrosome replication, which is independent of pRb/p107/p130 [21]. The resulting centrosome-related mitotic abnormalities are related to the occurrence of aneuploid genomes [21]. As an MT depolymerization protein, KIF2A plays a role in cancer progression. This study found that HPV16 E6/E7 protein can up-regulate the expression of KIF2A, which can regulate the motility of cervical cancer cells. We seem to find a new molecular mechanism that HPV16 E6/E7 protein participates in cervical cancer metastasis by regulating the depolymerization of MT.

Subsequently, using specific small-molecule inhibitors, we found that activation of downstream JNK signaling is required for E6/E7 to up-regulate KIF2A expression. The findings of Morgan et al. support our results. They found that HPV E6 oncogene induces JNK phosphorylation in the form of PDZ binding motifs, activates JNK/c-Jun signals, thereby regulating the invasion of cervical cancer cells [13]. However, their study did not show how activated JNK signaling regulates cell motility, whereas ours innovatively identified a role for KIF2A in this. Furthermore, their study used HeLa and CaSKi cell lines, which are different from those used in our study.

JNK is a stress-activated kinase belonging to the mitogen-activated protein kinase family. It was first discovered in 1990 and is able to phosphorylate Ser-63 and Ser-73 of c-Jun. Activation of JNK/c-Jun signaling plays a key role in the proliferation, apoptosis, invasion, chemosensitivity, and radiosensitivity of cervical cancer cells [22–26]. This study found that E6/E7 enhanced the phosphorylation of c-Jun by activating JNK signaling. p-c-Jun acts as a transcription factor to activate the transcription of KIF2A.

**Figure 5:**
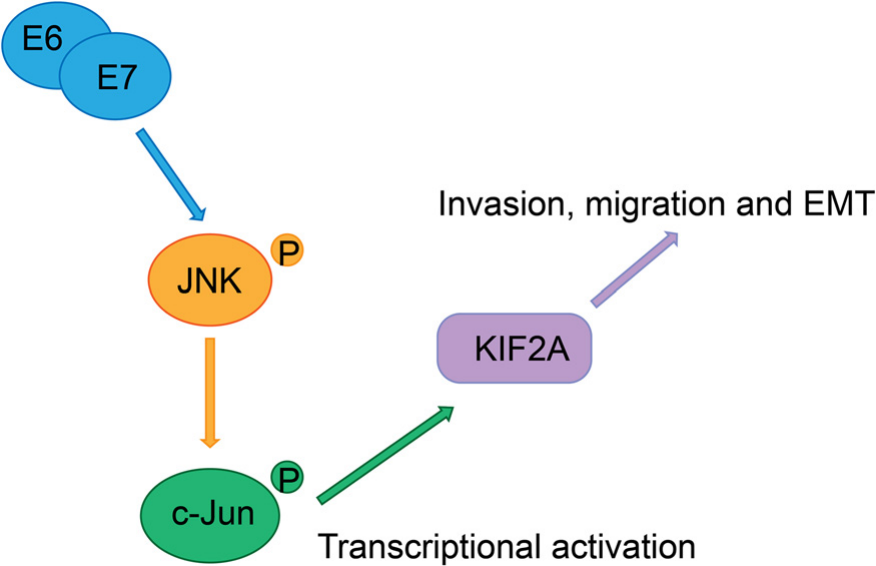
Signal pathways. KIF2A plays a key role in the invasion, migration, and EMT of cervical cancer cells. HPV16 E6/E7 can increase the levels of transcription factors c-Jun by activating the JNK signal, thereby up-regulating the transcriptional expression of KIF2A.

In conclusion, this study confirmed the role of KIF2A in cervical cancer cell motility, and found that E6/E7 oncogenes can activate the transcription of KIF2A by activating the JNK/c-Jun signaling pathway, thereby regulating cervical cancer cell migration and invasion (Figure 5). However, this study also lacked *in vivo* experimental validation and did not clarify the role of E6 and E7 alone.

## Abbreviations

DFS: disease-free survival
EMT: epithelial–mesenchymal transition
FIGO: Federation International of Gynecology and Obstetrics
HPV: human papillomavirus
JNK: c-Jun N-terminal kinases
MAPK: mitogen-activated protein kinase
MT: microtubules
OS: overall survival
SAPK: stress-activated kinase
